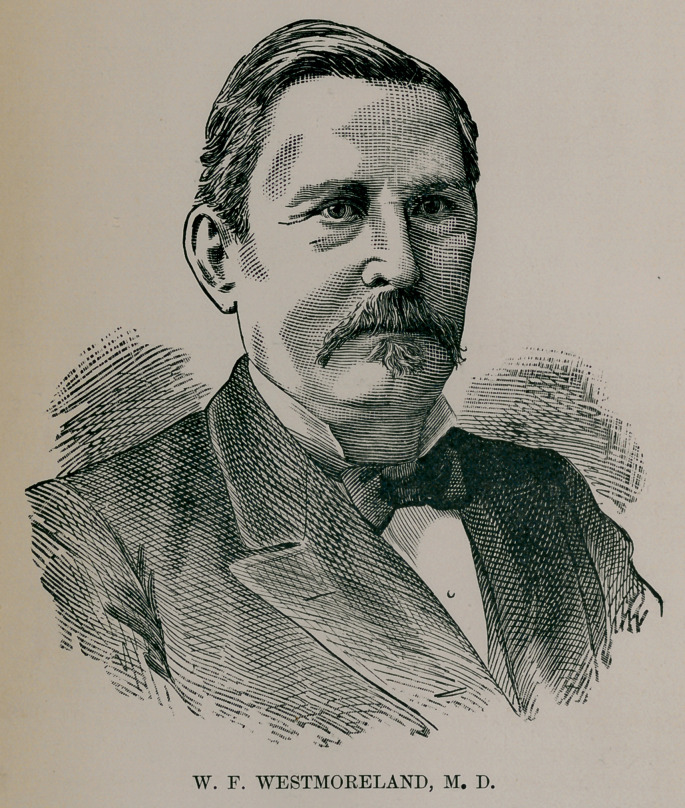# Our Portrait Gallery

**Published:** 1884-12

**Authors:** 


### OUR PORTRAIT GALLERY.

### WILLIS FOREMAN WESTMORELAND, M.D.

Dr. Westmoreland is a native of Fayette county, Georgia, where he was born January 1st, 1828. His father, Robert Westmoreland, was a planter of that county.

The Westmorelands of the United States have descended from three brothers, who, prior to the war for American independence, came to this country from England and settled respectively in the States of Pennsylvania, Virginia and North Carolina. The character of this family may be inferred from the fact that a county in each of these States bears their name. The subject of this sketch is a descendant of the North Carolina branch of the family. Having received primary instruction in a neighborhood school of his native county, his education was completed at a high school of Griffin, Georgia.

In 1847, impelled by the spirit of adventure and undecided as to his life occupation, he visited the State of Texas, mingling with the hardy inhabitants of that chivalric commonwealth, and familiarizing himself with the natural advantages of that section. He remained there for nearly twelve months, and about the close of that period he determined to devote himself to the profession of medicine, and returned to Georgia in order to enter upon the study of that science. In accordance with his nature, he promptly began and assiduously pursued his studies until prepared to enter a medical college with advantage and credit.

He attended his first course of lectures in the Medical College of Georgia at Augusta in 1848 and 1849. At the close of the latter year, he entered as a student Jefferson Medical College, Philadelphia, from which he graduated in 1850, with a class of which Dr. William H. Pancoast, now Professor of Anatomy in that college, Dr. S. Wier Mitchell, the celebrated Specialist of Philadelphia, and other eminent physicians were members.

Returning to Georgia he commenced the practice of medicine in his native county, but in July, 1851, moved to this city, where he prosecuted a successful and profitable practice until the fall of 1852. At this period he placed himself under the special instruction of Dr. Paul F. Eve, then Professor of Surgery in the Medical Department of the University at Nashville, Tennessee. There he remained for eight months, enjoying most excellent opportunities for acquiring knowledge in this branch of his profession.

Resolved to become master of surgery, he sailed for Europe in the winter of 1852. Arriving at Paris, he became at once an attendant upon the lectures of Valpeau, Nelaton, Rioux, Ricord and other eminent men of the profession, and applied himself assiduously to the details of that most delicate and responsible branch of medical science. He remained in Paris for about two years, enjoying the instruction of the most distinguished professors of Europe and opportunities for acquiring practical knowledge of surgery which were unsurpassed on the Continent. Appreciating these advantages, they were improved by him to the utmost.

In 1854, while he was in Paris, he was chosen Professor of Surgery in the Atlanta Medical College. He accepted the appointment, and returning home entered promptly and earnestly upon the duties of the position.

In 1855–6, he delivered a course of lectures on surgery in the Atlanta Medical College, which demonstrated his thorough knowledge of this branch of the profession, establishing at the same time a reputation for great skill as a surgeon by the successful performance of many delicate and difficult operations.

Impressed with the importance of a reliable medical journal in the South, he founded this journal during the summer of 1855. Until the year 1877, he continued his connection with this enterprise, either in the relation of editor or proprietor. Although he had acquired an enviable distinction as a surgeon, he aspired to higher attainments and still greater skill.

To gratify this laudable ambition he again sailed for Europe in September, 1856. As on his previous visit, he located in the French metropolis, and attended the lectures of the most eminent physicians and surgeons of that country. In addition to his attendance upon the lectures of these distinguished Professors, he became a private pupil of Dumas, the celebrated Oculist, Robin, the Microscopist, and Verneille, Surgical Pathologist, embracing in the scope of his studies and special instructions, everything auxiliary to a complete knowledge of Surgery.

In 1857, he returned to this State, bringing with him as trophies of his assiduous study, certificates of proficiency from the learned professors mentioned. Locating in Atlanta, he entered at once upon the practice of surgery, his renown as a surgeon constantly growing until his fame extended over the entire country.

The war of the States came on and, relinquishing a large and lucrative practice, he tendered his services to his native section. These were promptly and cordially accepted. As surgeon in the field, his knowledge and skill proved invaluable to the Southern army. Faithful, efficient and patriotic, he enjoyed the fullest confidence and esteem of those in power and command, while many a hero who bled for the Southern cause, realized his skillful and kindly ministrations on the field of conflict. Wherever ordered he went with alacrity, considering only the will of his country. Devoted to the cause of the Southern Confederacy, he followed her fortunes with pride and hope, until the last day of conflict when, at Appomatox, her flag was furled forever and the cause for which her heroes had battled was lost.

Turning sadly homeward, in sympathy with the people of the

South, he in due time arrived in this city, desolated by the merciless invader, and resumed the practice of his profession.

Although thorough in every branch of the healing science, and eminent as a general practitioner of medicine, he has, nevertheless, been particularly devoted to the practice of surgery. His successes in this branch of the profession have been wonderful and have made him a reputation that extends to every State of the Republic, classing him with the comparatively small number of our race who have fully achieved the purposes of their ambition.

All along the years of his professional life he has contributed by his pen to the advancement of medical science, so that he will live on in the light he has cast upon the theory and practice of healing. He lives as a friend of humanity, and when he shall have passed away from life, he will be cherished in memory as a true benefactor of his race.

Still actively engaged in the practice of surgery, and gathering new laurels at every step, he has a prospective career of greater usefulness. As President of the Faculty of the Atlanta Medical College, he is doing much to preserve and promote the prosperity and beneficial influence of that institution. His record is one in which his friends feel a just pride.

## Figures and Tables

**Figure f1:**